# Draft Genome Sequence of Bacillus selenatarsenatis SF-1^T^, a Promising Agent for Bioremediation of Environments Contaminated with Selenium and Arsenic

**DOI:** 10.1128/genomeA.01466-14

**Published:** 2015-01-22

**Authors:** Masashi Kuroda, Hiroyuki Ayano, Kazunari Sei, Mitsuo Yamashita, Michihiko Ike

**Affiliations:** a Division of Sustainable Energy and Environmental Engineering, Graduate School of Engineering, Osaka University, Yamadaoka, Suita, Osaka, Japan; b Department of Health Science, School of Allied Health Sciences, Kitasato University, Kitasato, Sagamihara-Minami, Kanagawa, Japan; c Department of Applied Chemistry, Faculty of Engineering, Shibaura Institute of Technology, Toyosu, Koto-ku, Tokyo, Japan

## Abstract

Bacillus selenatarsenatis sp. nov. strain SF-1^T^ is a promising agent for bioremediation of environments contaminated with selenium and arsenic. Here, we report the draft genome sequence of this strain.

## GENOME ANNOUNCEMENT

Selenium is a minor metal used as a raw material for the production of semiconductors, photovoltaic cells, etc. It is a known toxic contaminant of the aquatic environment when present in soluble, oxidized forms as selenate and selenite. Conversely, arsenic contamination in drinking water is a serious problem plaguing several countries. Soil contaminated with arsenic due to anthropogenic or nonanthropogenic reasons increases health risk due to leaching of arsenic into ground and surface water.

Strain SF-1^T^, the type-strain of a novel bacterial species Bacillus selenatarsenatis (1) isolated from sediments obtained from a drain receiving effluents from a glass manufacturing plant (2), is believed to be a promising agent for bioremediation of environments contaminated with selenium and arsenic (2, 3). Strain SF-1^T^ reduces selenate to selenite through anaerobic respiration, and subsequently into elemental selenium (2). The insoluble elemental selenium particles can be readily removed from the water phase, enabling the treatment of selenate/selenite-containing water. Strain SF-1^T^ also reduces arsenate to arsenite through anaerobic respiration (3). The reduction of arsenate, which is strongly absorbed by soil, to soluble arsenite can facilitate the removal of arsenic from contaminated soil (4). The molecular mechanisms of these reductions have not yet been elucidated, except for the respiratory reduction of selenate catalyzed by membrane-bound selenate reductase, SrdBCA (5).

The genomic DNA of strain SF-1^T^ was extracted as previously described (6). Initially, the sequencing was performed by a 454 Genome Sequencer 20 (Hoffmann-La Roche, Basel, Switzerland). Further sequencing was conducted using a Roche 454 Genome Sequencer FLX (Hoffmann-La Roche). A total of 80,565,561 bp reads were obtained. The reads were assembled using Newbler version 2.3 into a draft genome sequence. The draft sequence consisted of 145 contigs, a G+C content of 42.1% for a total of 4,756,255 bp, an *N*_50_ statistic of 58,218 bp, and a maximum contig size of 147,506 bp. A total of 5,011 coding sequences (CDSs) were predicted by the RAST Prokaryotic Genome Annotation Server (7).

The *srdBCA* operon was found in the contig BSSF00031. The region comprising a putative arsenic resistant (*ars*) operon and a respiratory arsenate reductase operon (*arr*) (8) in contig BSSF00009 was predicted to be responsible for arsenic metabolism. Most CDSs were annotated with genes from a variety of bacteria belonging to the family Bacillaceae, without any bias for a specific genus, highlighting the taxonomic uniqueness of B. selenatarsenatis. This draft genome data may provide a basis for the study of bacterial anaerobic respiration, as well as bioremediation of polluted environments.

### Nucleotide sequence accession numbers.

The draft genome sequence and annotations were deposited in the DNA Data Bank of Japan (DDBJ) under the accession numbers BASE01000001 to BASE01000145.
